# Different Achilles Tendon Pathologies Show Distinct Histological and Molecular Characteristics

**DOI:** 10.3390/ijms19020404

**Published:** 2018-01-30

**Authors:** Franka Klatte-Schulz, Susann Minkwitz, Aysha Schmock, Nicole Bormann, Alper Kurtoglu, Serafeim Tsitsilonis, Sebastian Manegold, Britt Wildemann

**Affiliations:** 1 Julius Wolff Institute, Center for Musculoskeletal Surgery, Charité—Universitätsmedizin Berlin, Corporate Member of Freie Universität Berlin, Humboldt-Universität zu Berlin, and Berlin Institute of Health, 13353 Berlin, Germany; Susann.Minkwitz@charite.de (S.Mi.); Aysha.Schmock@charite.de (A.S.); Nicole.Bormann@charite.de (N.B.); Alper.Kurtoglu@charite.de (A.K.); Serafeim.Tsitsilonis@charite.de (S.T.); Sebastian.Manegold@charite.de (S.Ma.); Britt.Wildemann@charite.de (B.W.); 2 Berlin-Brandenburg Center for Regenerative Therapies, Charité—Universitätsmedizin Berlin, 13353 Berlin, Germany

**Keywords:** chronic tendon pathologies, acute rupture, human, tendon structure, matrix, modeling/remodeling, inflammation, fat, innervation

## Abstract

Reasons for the development of chronic tendon pathologies are still under debate and more basic knowledge is needed about the different diseases. The aim of the present study was therefore to characterize different acute and chronic Achilles tendon disorders. Achilles tendon samples from patients with chronic tendinopathy (*n* = 7), chronic ruptures (*n* = 6), acute ruptures (*n* = 13), and intact tendons (*n* = 4) were analyzed. The histological score investigating pathological changes was significantly increased in tendinopathy and chronic ruptures compared to acute ruptures. Inflammatory infiltration was detected by immunohistochemistry in all tendon pathology groups, but was significantly lower in tendinopathy compared to chronic ruptures. Quantitative real-time PCR (qRT-PCR) analysis revealed significantly altered expression of genes related to collagens and matrix modeling/remodeling (matrix metalloproteinases, tissue inhibitors of metalloproteinases) in tendinopathy and chronic ruptures compared to intact tendons and/or acute ruptures. In all three tendon pathology groups markers of inflammation (*interleukin* (*IL*) *1β*, *tumor necrosis factor α*, *IL6*, *IL10*, *IL33*, *soluble ST2*, *transforming growth factor β1*, *cyclooxygenase 2*), inflammatory cells (*cluster of differentaition* (*CD*) *3*, *CD68*, *CD80*, *CD206*), fat metabolism (*fatty acid binding protein 4*, *peroxisome proliferator-activated receptor γ*, *CCAAT/enhancer-binding protein α*, *adiponectin*), and innervation *(protein gene product 9.5*, *growth associated protein 43*, *macrophage migration inhibitory factor*) were detectable, but only in acute ruptures significantly regulated compared to intact tendons. The study gives an insight into structural and molecular changes of pathological processes in tendons and might be used to identify targets for future therapy of tendon pathologies.

## 1. Introduction

Achilles tendon pathologies can either be due to an acute injury, mostly occurring in relation to sports, or have a chronic background and are called tendinopathies. Achilles tendinopathy is one of the most frequent tendon pathologies caused by overuse or overload stresses, which lead to repetitive micro-traumata [1]. It is characterized by swelling, pain and reduced function [2] and was supposed to represent a failed healing response of the tendon [3]. Characteristics of tendinopathic tendons are excessive proliferation of tenocytes and changes in tenocyte morphology, disruption of collagen fibers, increase in non-collagenous matrix such as glycosaminoglycans (GAG) and fat tissue as well as neovascularization [4,5,6,7,8,9]. Furthermore, it was reported that alterations in the expression of matrix metalloproteinases (MMPs) and their natural antagonists, the tissue inhibitors of metalloproteinases (TIMPs), which regulate the modeling and remodeling in the tendons, are linked to tendinopathy [10,11,12,13]. The presence of immune cells and inflammatory processes in tendinopathy is still under debate [14,15,16,17]. Several studies using classical histological evaluations negate the presence of immune cells in chronic tendinopathy [2,9,18]. On the other hand, more recent studies found inflammatory infiltration of B cells, T cells, macrophages, mast cells and/or natural killer cells [19,20,21,22,23,24]. Furthermore, it was hypothesized that inflammation is only relevant in the initiation of tendinopathy rather than the progression of the disease process [25]. It was shown that chronic painful tendons are associated with an increase in neuromediators like the neurotransmitter glutamate and glutamineric receptors, substance P and protein gene product 9.5 (*PGP9.5*) [26,27,28,29], as well as an increase in inflammatory markers such as cluster of differentiation (CD)206 and CD45 [27]. Additionally, it was shown that the pro-inflammatory cytokines cyclooxygenase-2 (*COX2*) and interleukin-6 (*IL6*) were upregulated in painful versus asymptomatic Achilles tendons [30].

Taken together it seems obvious that tendinopathy is a multifactorial process and more basic knowledge is still needed to better understand the development and progression of the disease. Therefore, the present approach aimed to characterize different tendon pathologies, such as tendinopathy and chronic Achilles tendon ruptures compared to acute ruptures and intact tendons regarding molecular and histological alterations in matrix production, modeling and remodeling, adipogenic differentiation, as well as inflammation and innervation.

## 2. Results

### 2.1. Histological Analysis

**Figure 1:**
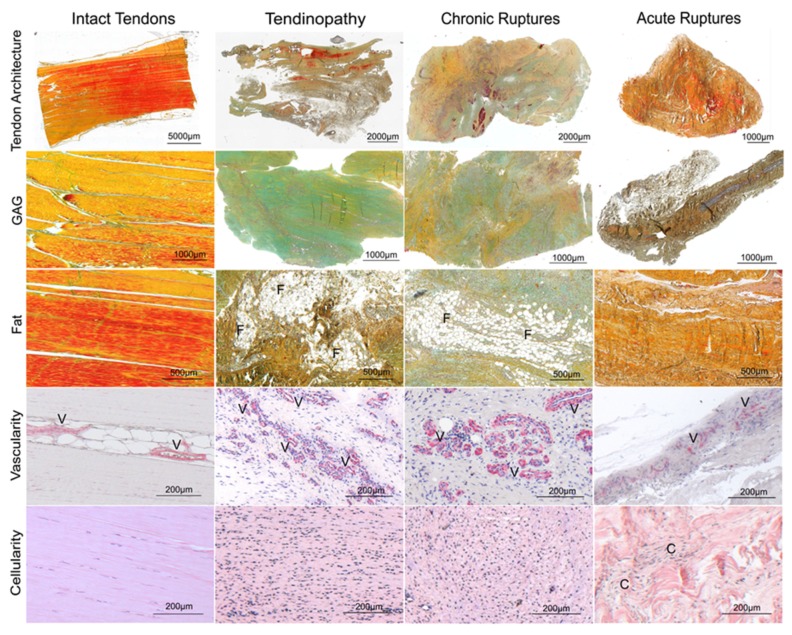
Exemplary histologies of intact tendons, tendinopathic tendons, chronic ruptures and acute ruptures using Movat Pentachrome (MP) staining (tendon architecture, glycosaminoglycan (GAG) and fat tissue (lipid vacuoles)), α smooth muscle actin (αSMA) staining (vascularity) and Hematoxylin Eosin (HE) staining (cellularity). F: fat tissue, V: vessels, C: cell clusters. The Movat Pentachrome staining used stains collagen in yellow to brown, mature collagen in red [31], GAG ground substance in turquois, cell nuclei in blue to black and cytoplasm stains reddish.

**Figure 2:**
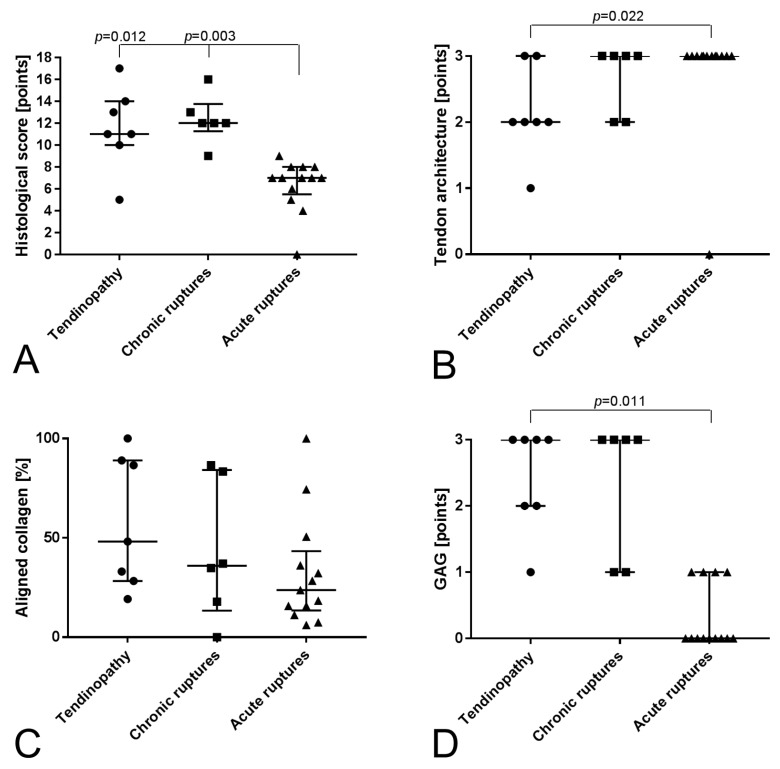

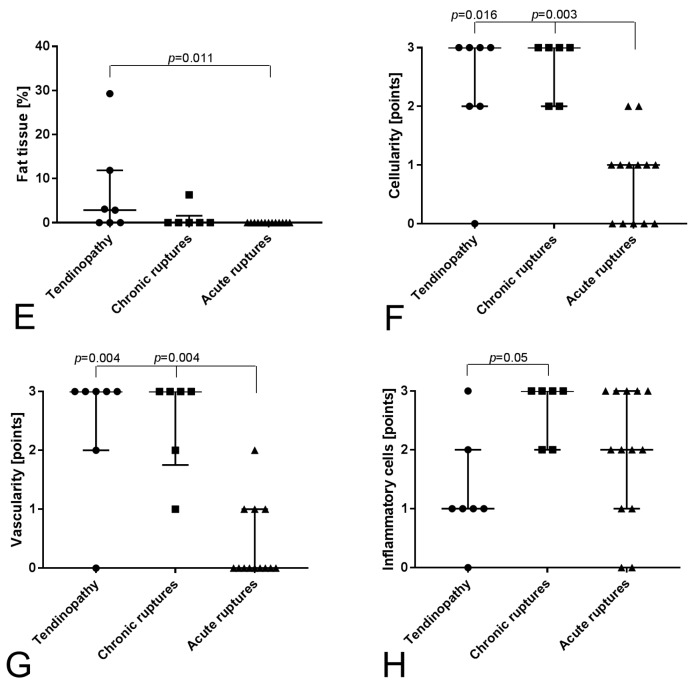
Evaluation of histological score: (**A**) Total histological score reaching 18 points as maximum, which would indicate a highly degenerated tendon. (**B**) Tendon architecture as indicator for the disturbance of the tendon structure (0–3 points). (**C**) Amount of aligned collagen quantified using ImageJ (0–100%). (**D**) GAG content (0–3 points, MP staining). (**E**) Fat tissue quantified using ImageJ (0–100%, MP staining). (**F**) Cellularity (0–3 points, HE staining). (**G**) Vascularity (0–3 points, αSMA staining). (**H**) Inflammatory cells (not included in histological score, CD45 staining). Data are given as individual dot plots with median and interquartile range.

**Figure 3:**
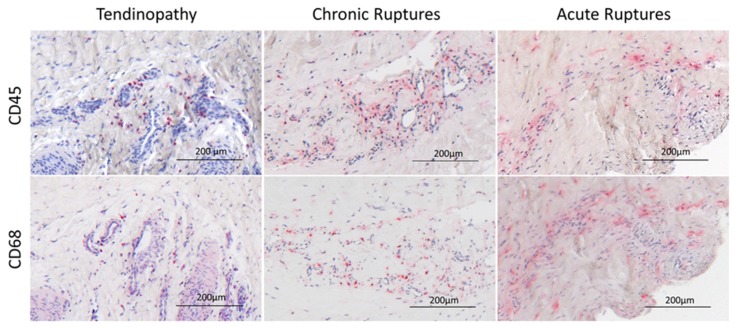
Exemplary images of inflammatory cell infiltration visualized by immunohistological CD45 (leucocytes) and CD68 (monocytes/macrophages) staining in tendinopathic tendons, chronic ruptures and acute ruptures.

The intact cadaveric tendons showed compact collagen fiber bundles with long flat tenocytes (Figure 1) and were all scored with zero points. The tendinopathy and chronic rupture groups showed a significantly increased histological score compared to the acute ruptured tendons (Figure 2A). Looking in detail, the tendon architecture was fully disturbed in all acute ruptured tendons, which was significantly higher scored compared to the tendinopathic tendons (Figure 1 and Figure 2B). The amount of aligned collagen did not significantly change between the groups (Figure 2C). In the tendinopathy and chronic ruptures, a high GAG content was detected (Figure 1). The difference was significant in the tendinopathy group compared to the acute ruptures (Figure 2D). Fat infiltration was seen in four tendinopathy tendons and in two chronic ruptures and was significantly higher in the tendinopathic tendons compared to the acute ruptures, where no fat tissue was visible (Figure 1 and Figure 2E). The tendinopathy and chronic ruptures were histologically characterized by a tightly packed structure with significantly higher vascularity and hypercellularity. In acute ruptured tendons, vessel formation was low and mostly concentrated at the tendon edges, whereas in intact tendons vessels were only present between the collagen fiber bundles (Figure 1 and Figure 2F,G). A significantly lower amount of inflammatory cells (CD45^+^) was found in the tendinopathy group compared to the chronic ruptures. High amounts of inflammatory cells were also found in acute ruptured tendons (Figure 2H). The CD45^+^ cells were primarily identified as monocytes/macrophages (CD68^+^) (Figure 3).

### 2.2. Gene Expression

**Figure 4:**
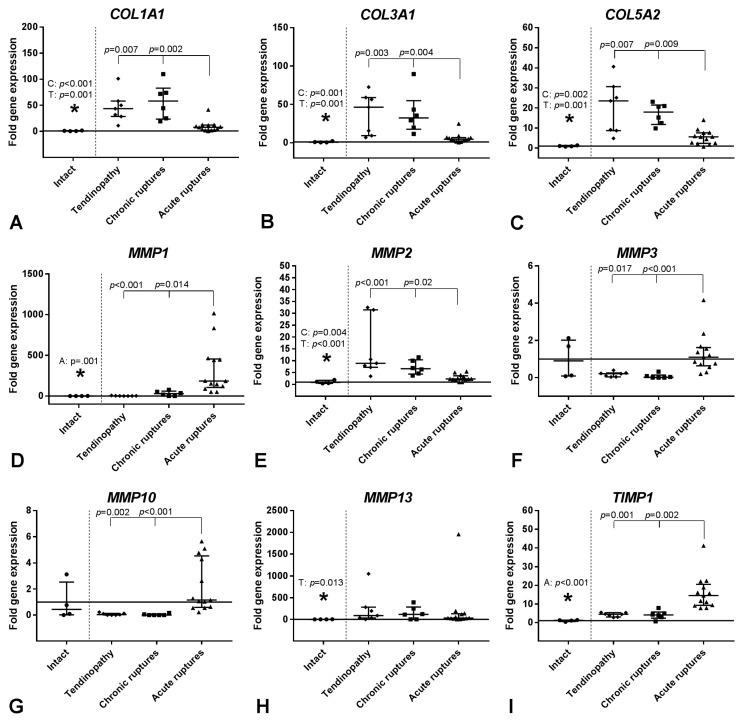

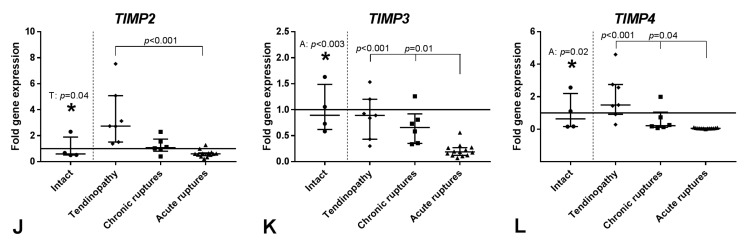
Relative gene expression of collagens (**A**–**C**), MMPs (**D**–**H**) and TIMPs (**I**–**L**) in tendinopathic tendons, chronic ruptures and acute ruptures given as fold to intact tendon tissue (horizontal line, mean value = 1). qRT-PCR data were normalized to the expression of the house keeping gene *18S rRNA* with efficiency correction and presented as fold change to the mean of the intact tendons. Results are shown as individual dot plots with median and interquartile range. Significant differences between the intact tendons and the three tendon pathology groups are marked with a star (*) above the intact tendon and the respective significant group (T: tendinopathy, C: chronic ruptures, A: acute ruptures) and the *p*-value. The vertical dashed line separates the intact group from the tendon pathology groups. Significant differences between the three tendon pathology groups are marked with a spanning line above the groups and the individual *p*-values. *p* ≤ 0.05 was considered as statistically significant.

Using quantitative real-time PCR (qRT-PCR), the collagens *Col1A1*, *Col3A1* and *Col5A2* were found to be significantly upregulated in the tendinopathies and chronic ruptures compared to the intact control and the acute ruptured tendons (Figure 4A–C). The *Col1A1/Col3A1* ratio was not regulated.

When looking at matrix degrading enzymes, the gene expression of the collagenase *MMP1* was significantly elevated in the acute ruptured tendons compared to all other groups, whereas the expression of the collagenase *MMP13* was significantly increased in the tendinopathy group compared to the intact samples (Figure 4D,H). The gelatinase *MMP2* was significantly upregulated in the tendinopathy and chronic rupture group compared to the intact and acute ruptured tendons (Figure 4E), but *MMP9* was not affected. The expression of the stromelysines *MMP3* and *MMP10* was significantly decreased in the tendinopathy and chronic rupture groups compared to the acute ruptured tendons (Figure 4F,G).

The expression of the MMP inhibitors was differentially regulated. *TIMP1* expression was significantly higher and *TIMP3* and *TIMP4* expression significantly lower in the acute ruptured tendons compared to the other groups. *TIMP2* expression was significantly elevated in the tendinopathy group compared to the intact tendons and acute ruptures (Figure 4I–L).

**Figure 5:**
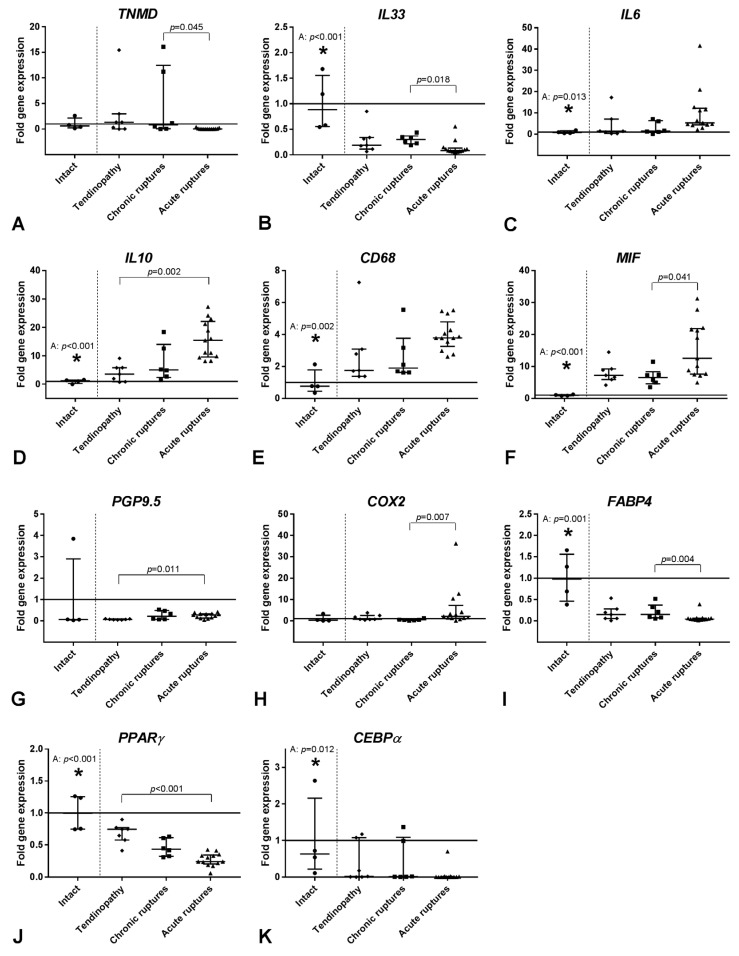
Relative gene expression of *TNMD* (**A**), the inflammatory cytokines *IL33*, *IL6* and *IL10* (**B**–**D**), the immune cell marker *CD68* (**E**), the nerve markers *MIF* and *PGP9.5* (**F**–**G**), the pain marker *COX2* (**H**), and the fat markers *FABP4*, *PPARγ* and *CEBPα* (**I**–**K**) in tendinopathic tendons, chronic ruptures and acute ruptures given as fold to intact tendon tissue (horizontal line, mean value = 1). qRT-PCR data were normalized to the expression of the house keeping gene *18S rRNA* with efficiency correction and presented as fold change to the mean of the intact tendons. Results are shown as individual dot plots with median and interquartile range. Significant differences between the intact tendons and the three tendon pathology groups are marked with a star (*) above the intact tendon and the respective significant group (T: tendinopathy, C: chronic ruptures, A: acute ruptures) and the *p*-value. The vertical dashed line separates the intact group from the tendon pathology groups. Significant differences between the three tendon pathology groups are marked with a spanning line above the groups and the individual *p*-values. *p* ≤ 0.05 was considered as statistically significant.

The tendon related transcription factor scleraxis (*SCX*) was not significantly altered between the four groups. The tendon related proteoglycan tenomodulin (*TNMD*) was significantly upregulated in chronic versus acute ruptured tendons (Figure 5A).

When looking at inflammatory markers, the *IL33* expression was significantly decreased in the acute ruptures compared to the intact tendons and chronic ruptures (Figure 5B). The IL33 receptor soluble ST2 (*sST2*) was not regulated between the groups . In the group of pro- and anti-inflammatory cytokines only *IL6* and *IL10* showed significant upregulation in the acute ruptures compared to the intact and/or tendinopathic tendons (Figure 5C,D), whereas the expression of *IL1β*, tumor necrosis factor α (*TNFα*) and transforming growth factor β1 (*TGFβ1*) did not change between the groups.

The expression of the T-cell marker *CD3* was not regulated, but the expression of the monocyte/macrophage marker *CD68* was significantly upregulated in the acute ruptures compared to the intact tendons (Figure 5E). The macrophage polarization markers *CD80* and *CD206* did not significantly change.

The expression of macrophage migration inhibitory factor (*MIF*), a nerve regeneration marker, and *PGP9.5*, a general nerve marker, was significantly increased in acute ruptures compared to the intact and chronic ruptures or tendinopathy group, respectively (Figure 5F,G). The expression of the nerve growth marker growth associated protein 43 (*GAP43*) was not regulated. The expression of the pain-associated factor *COX2* was significantly increased in the acute ruptures compared to the chronic ruptures (Figure 5H).

When looking at the fat metabolism, significantly lower expression of fatty acid binding protein 4 (*FABP4*), peroxisome proliferator-activated receptor γ (*PPARγ*), and CCAAT/enhancer-binding protein α (*CEBPα*) was seen in acute ruptures compared to the intact tendons. On the other hand, *FABP4* and *PPARγ* expression was significantly higher in chronic ruptures and tendinopathic tendons when compared to acute ruptures (Figure 5H,I). The expression of adiponectin (*ADIPOQ*) was not significantly affected.

## 3. Discussion

The present study aimed at investigating the structural, cellular, as well as molecular characteristics of different tendon pathologies. As tendinopathy seems to be a multifactorial process, a variety of factors and processes related to tendon matrix, modeling and remodeling, inflammation, fatty infiltration and innervation were analyzed to better understand possible reasons for the development and progression of degenerative Achilles tendon pathologies.

The tendinopathy and chronic rupture groups showed a disturbed tendon structure and were histologically characterized by a tightly packed extracellular matrix (ECM) with loss of the hierarchical structure, high amounts of non-collagenous matrix such as GAG and fat, high vascularity and hypercellularity, as it was reported previously [4,5,6,7,8,9]. Interestingly, despite the different pathomechanisms of the two degenerative tendon pathologies, which is on the one hand the overused tendon with repetitive micro-traumata (tendinopathy) and on the other hand the tendons with overseen or old macro-trauma (chronic ruptures), both groups showed similar characteristics. Only the lower amount of inflammatory cell infiltration (CD45^+^) in the tendinopathy group was significantly different compared to the chronic ruptures. Additionally, the tendinopathy group showed more distinct differences than the chronic ruptures compared to the intact and acute ruptured tendons. Overall, the acute ruptured tendons showed the strongest alterations compared to the intact tendons for nearly all analyzed processes (modeling/remodeling, inflammation, immune cells, fatty infiltration and innervation), whereas the tendinopathy and chronic ruptured tendons only showed significant differences to the intact controls related to collagen and matrix modeling/remodeling.

The disturbed matrix metabolism as seen in the histology in the tendinopathy and chronic ruptured tendons was displayed in the altered gene expression of the collagens as well as MMPs and TIMPs. The expression of *COL1A1*, *COL3A1* and *COL5A2* was significantly elevated compared to intact and acute ruptured tendons. Accordingly, this increase in collagen expression was also reported by other authors [12,30,32]. An increase in type III and V collagen expression was often reported to result in inferior biomechanical properties [33,34]. Furthermore, type V collagen variants were associated with chronic Achilles tendinopathy [35,36]. The loss of ability to form a hierarchical tendon structure seems to be a further important characteristic in tendinopathy. *TNMD*—a tendon proteoglycan responsible for collagen fiber maturation [37,38]—was significantly upregulated in the chronic rupture group compared to acute ruptures and not regulated in the tendinopathy group. Non-regulation of factors regulating fibrillogenesis were also reported previously for tendinopathy versus healthy tendons [32], which partly fits with the present findings. IL33—a cytokine having a pivotal role in innate and acquired immune responses—was described to be important in early tendinopathy. Here it regulates the transition of collagen type I to type III synthesis through its cognate receptor ST2, which exists in a membrane-bound as well as soluble cognate form (sST2) [39]. Presently, *IL33* expression was significantly decreased in acute ruptured versus intact tendons but significantly increased in chronic ruptures compared to acute ruptures. This is supporting the previous hypothesis that recurrent micro-traumata lead to an increase in *IL33* expression and thus matrix degradation and inflammation, which might lead to an impaired healing response [39] and possibly to a lower tendon strain and a higher susceptibility for Achilles tendon rupture.

Next to the collagens, matrix degrading enzymes were also regulated in the tendinopathy and chronic ruptured tendons. The expression of the gelatinase *MMP2* was significantly increased in both groups, which is in accordance to previous findings [32]. Additionally, the collagenase *MMP13* expression was significantly upregulated in the tendinopathy group compared to the intact tendons. The upregulation of the MMPs together with the mostly non-regulation of the TIMPs compared to the intact tendons might lead to a possible MMP/TIMPs imbalance in the tendinopathy and chronic rupture groups. Such an imbalance was hypothesized to lead to a failed tendon remodeling process [40,41,42]. Additionally, the expression of *MMP3* and *MMP10*, the stromelysines, which have an important regulatory function on other MMPs [42], was significantly decreased in the tendinopathy and chronic rupture groups compared to the acute ruptured tendons. A decrease of *MMP-3* expression was also reported by others and was hypothesized to lead to an incorrect remodeling process [12,42]. Present regulations regarding MMPs and TIMPs were previously also shown for tenocytes from aged donors and with higher degenerative status of supraspinatus tendons [43]. Additionally, comparable regulations were found in acute ruptured tendons, which were surgically treated at a later time-point (7–10 days) post rupture compared to tendons, were the surgery was performed at an earlier time-point (2–4 and 5–6 days) after Achilles tendon rupture [44]. As hypothesized previously, at a later time point the tendons seem to turn into a degenerative direction, which can now be confirmed when comparing it to the present tendinopathy and chronic rupture groups.

Interestingly, no significant changes for the tendinopathy and chronic rupture group compared to intact tendons could be observed for the other processes such as inflammation, fatty infiltration and innervation. An excessive inflammatory reaction was not present in the tendinopathy group and was even reduced, as seen by a lower amount of inflammatory cells in the tendinopathy group compared to the chronic ruptures in the histology. Inflammation is present in tendinopathy and chronic ruptured tendons but seems to be less pronounced compared to the acute ruptured tendons, as the expression of pro-and anti-inflammatory cytokines as well as inflammatory cell markers for T-cells and macrophages was not distinctly different compared to the expression in intact tendons. The presence of inflammatory cell infiltration in tendinopathy and chronic ruptures confirms several previous studies [19,20,21,22,23,24], but the role of inflammation in the disease is still unclear but might only be important in the initiation of tendinopathies [25]. However, inflammation seems to be more important in acute ruptures, as indicated by the increased expression of the cytokines *IL6* and *IL10* and the macrophage marker *CD68*. The increased expression of *IL6* and *CD68* might be linked to each other, as IL6 was reported to promote the differentiation of monocytes into macrophages [45]. An increased *IL6* expression was also found by others in painful and ruptured versus asymptomatic tendons [30]. Interestingly, IL6^−/−^ mice showed inferior mechanical properties [46]. During acute tendon healing, the typical processes such as inflammation (pro- and anti-inflammatory phase), proliferation, remodeling and maturation proceed [47]. The present data suggest that in the acute ruptured tendons, which were taken four to six days after rupture, the healing process is in the anti-inflammatory phase, as both IL6 and IL10 have anti-inflammatory properties [48] and the pro-inflammatory cytokines *IL1β* and *TNFα* were not regulated. This is furthermore underlined by unregulated macrophage polarization (unchanged *CD80*, *CD206* expression), as M1 macrophages have pro- and M2 macrophages have anti-inflammatory properties [49]. However, macrophage polarization was previously described to be involved in tendinopathy, with M2 macrophages or *CD206* expression is dominating in chronic tendon pathologies [24,50]. Additionally, we could show previously that tenocytes respond to an inflammatory environment and influence macrophage polarization [51]. Presently, no regulation in the expression of macrophage polarization markers could be observed, either in acute ruptures nor in tendinopathy or chronic ruptured tendons, which might be caused by limitations in the used methods.

A clear fatty infiltration was detected in the histological sections for tendinopathy and partly chronic tendon ruptures. However, no significant differences between gene expression levels of the adipogenic factors *FABP4*, *PPARγ* and *ADIPOQ* were detected between tendinopathy and chronic ruptures with intact tendons. The presence of adipogenic factors in intact tendons is not unusual, as isolated tenocytes of supraspinatus tendons also expressed adipogenic factors without adipogenic stimulation [52,53,54]. Additionally, others found a reduced expression of markers for lipolysis and *ADIPOQ* and an increase in markers for fatty acid β-oxidation in Kargers fat pad in patients with Achilles tendinopathy, which was discussed as being a paradox but might be caused by different regulation of mRNA expression and actual enzyme activity [55]. However, presently the increase in fat tissue in the tendinopathy and chronic ruptured tendons compared to the acute ruptured tendons was confirmed by the gene expression data, with an increased *FABP4* and *PPARγ* expression in tendinopathy and chronic ruptured tendons. It was previously shown that adipose tissue plays an important role in inflammation [55,56] and that ADIPOQ might have pro-inflammatory properties in joint diseases [57]. However, the present results were not able to confirm a relationship between adipose tissue and inflammation in tendinopathy and chronic ruptures.

Innervation could be demonstrated in the present study, with expression of the general nerve marker *PGP9.5*, the nerve growth marker *GAP43* and the nerve regeneration marker *MIF* [58]. PGP9.5 was previously found in the plantaris tendon and peritendinous tissue of patients with Achilles tendinopathy, indicating strong innervation [28] and was additionally associated with pain [27]. *MIF* expression was not described in tendons before and no significant differences in expression were observed between tendinopathy/chronic ruptures and intact tendons. However, innervation seems to play a more important role in the acute situation, as acute ruptured tendons expressed higher amounts of *PGP9.5* and *MIF* compared to tendinopathy, and chronic/intact tendons, respectively. The further pain-associated factor *COX2* was upregulated in acute ruptured tendons compared to chronic ruptured tendons. An increased *COX2* expression was previously found in painful Achilles tendons but even more in acute ruptured Achilles tendons [30], which underlines the present findings.

### Limitations

Most regulations were observed for the acute ruptured tendons, which might be caused by the higher sample number in the group. The sample number is relatively low for the intact, tendinopathy and chronic rupture group, which might account for the less distinct regulations. To increase the sample number for intact tendons another tendon location could have been used, as performed in other studies [24,59]. However, we decided to use the same tendon as control to avoid regulations due to a different biomechanical stimulation of the tendons and surrounding tissues. The intact Achilles tendons derived from patients with indications for foot surgery not related to primary Achilles tendon pathology. Samples were taken during Achilles tendon lengthening with tendon transfer for peroneal paresis and implantation of total ankle arthroplasty. These indications might harm the normal movement of the foot and therefore also Achilles tendon, which could have affected the present results of the intact group.

Several analyzed factors and mechanisms are linked to each other, such as MMPs and cytokines, as MMPs also have non-ECM substrates such as IL1β and TNFα [60]. The present study is limited to a descriptive histological and molecular biological level as special mechanisms and interplays cannot be clarified. The available tendon material was limited and we decided to use a descriptive (histology) and quantitative (qRT-PCR) method to analyze the tendon samples in the most comprehensive way. A quantitative protein analysis to validate the expression data was not possible with the limited material.

Regarding the donor demographics, the age of the groups varied and donors with chronic ruptures were significantly older compared to donors with acute ruptures. As we showed previously that the age of donors can affect the biological characteristics of tenocytes of the rotator cuff [43,54], we are not able to fully exclude that changes between the two groups are partially affected by the varying donor age. However, for the acute rupture group we deliberately choose a cohort of young recreational sportsmen to avoid analyzing tendons with degenerative preconditions to better border them from the two chronic tendon pathology groups.

## 4. Materials and Methods

### 4.1. Tendon Sampling

Achilles tendon biopsies from patients suffering from insertional Achilles tendinopathy (*n* = 7) or chronic delayed diagnosed ruptures (*n* = 6; with a trauma to surgery duration of ≥3 weeks) were taken at surgery. Additionally, acute ruptured Achilles tendons from recreational sportsmen, which were taken during minimal invasive Achilles tendon reconstruction 4 to 6 days (mean 4.9 ± 0.9 days) after rupture (*n* = 13) were used for comparison. Patients who received oral medication or local injections, which might influence tendon structure (systemic cortisone, antibiotics and anabolic steroids) were excluded. Intact tendons from patients undergoing surgery not related to primary tendon pathology served as control for RNA analysis (*n* = 4) and from cadavers for histological evaluations (*n* = 5). The donor demographics are summarized in Table 1. The samples of the intact human Achilles tendons as well as the acute ruptured tendons were partly used in a previous study [44]. The study was approved by the local institutional review board (EA2/074/14, 02 July 2014) and all patients gave their written informed consent prior to surgery. The tendon samples were divided into two equal parts and analyzed histologically and by qRT-PCR.

### 4.2. Histological Evaluation

Tendon samples were processed as published previously for histological evaluation [44]. They were fixed in 4% paraformaldehyde solution for 24 h until rinsing, automatic dehydration and paraffin embedding. Slices 4 µm thick were cut and stained with Hematoxylin Eosin (HE), Movat Pentachrome (MP) and the antibody against α-smooth muscle actin (αSMA). A modified Movin score [9] was used to evaluate pathologic changes in tendon structure as described previously [44]. The following parameters were evaluated: tendon architecture, amount of aligned collagen, GAG content, fat content, cellularity and vascularity. A total of 18 points could be reached with each parameter being scored between 0 and 3 (0 = normal, 1 = slightly abnormal, 2 = abnormal and 3 = markedly abnormal) (for classification, see [44]). The parameters were quantified visually by three blinded independent observers, whereas 2 observers independently scored the samples and one decided in case of discrepancy. Using ImageJ (ImageJ 1.44i, Wayne Rasband, National Institute of Health, Bethesda, MD, USA) the amount of fat tissue and aligned collagen was quantified. CD45 and CD68 immunohistochemical stainings were performed to detect inflammatory cell infiltration.

### 4.3. Immunohistochemistry

Immunohistochemical stainings were performed to analyze vessel formation (αSMA, monoclonal mouse anti-human smooth muscle actin, 1:200, Dako, Jena, Germany, M0851), as well as inflammatory cell infiltration (CD45, monoclonal mouse Anti-Human CD45, 1:100, Dako, M0701, and CD68, monoclonal mouse anti-human CD68, 1:40, Acris Antibodies GmbH, Herford, Germany). Primary antibodies were incubated for 1 h and stainings afterwards processed with the ZytoChem-Plus AP Kit (Broad Spectrum, AP060, Zytomed, Berlin, Germany) and the Alkaline Phosphatase Substrate Kit I (Sk-5100, Vector, Peterborough, UK). Mayer’s Hematoxylin (Sigma-Aldrich, Taufkirchen, Germany) was used for counterstaining. Isotype control and antibody only staining served as negative controls.

### 4.4. qRT-PCR

qRT-PCR was performed as described previously [44]. Tendon samples were incubated in RNAlater (Qiagen, Hilden, Germany) for transport and stored at −80 °C until used for RNA isolation. A liquid nitrogen cooled steel mortar system and Trifast peqGOLD (Peqlab, Erlangen, Germany) were used for tissue homogenization. Phase separation was performed using chloroform. RNA was purified with the NucleoSpin RNA Kit (Macherey-Nagel, Düren, Germany) according to the manufacturer and afterwards quantified with the Nanodrop ND1000 system (Peqlab, Erlangen, Germany). Exemplary analysis of RNA integrity was performed with the 2100 Bioanalyzer and the Agilent 6000 Pico Kit (Agilent, Santa Clara, CA, USA) and revealed good RNA quality with only slight RNA degradation (values between 6.7 and 6.9). cDNA synthesis of 100 ng RNA template was performed with the qScript cDNA Supermix (Quanta Biosciences, Gaithersburg, MD, USA). Expression levels of collagens (*Col1A1*, *Col3A1*, *Col5A2*), tendon markers (*SCX*, *TNMD*), matrix degrading enzymes (*MMP1*, *MMP2*, *MMP3*, *MMP9*, *MMP10*, *MMP13*) and its inhibitors (*TIMP1*, *TIMP2*, *TIMP3*, *TIMP4*), pro- and anti-inflammatory cytokines (*IL1β*, *TNFα*, *IL6*, *IL10*, *TGFβ1*, *COX2*, *IL33*, *sST2*), immune cell markers (*CD3* (T-cells) and *CD68* (monocytes/magrophages)), macrophage polarization markers (*CD80* (M1), *CD206* (M2)), markers for adipogenic differentiation (*FABP4*, *PPARγ*, *ADIPOQ*, *CEBPα*) as well as markers for innervation (*PGP9.5*, *GAP43*, *MIF*) were evaluated. The *18S rRNA* was chosen as housekeeping gene because it was most constant compared to hypoxanthine phosphoribosyl transferase (HPRT) and 60S ribosomal protein L13 (RPL13). The SYBR Green Mastermix (Quanta Biosciences) and the LightCycler 480 System (Roche, Mannheim, Germany) were utilized to perform qRT-PCR. Primer 3 software was used to design all primer sequences (Freeware; Available online: http://frodo.wi.mit.edu/primer3) except for *TNMD* [61] and *IL1β* [62]. Primer sequences were produced by Tib Molbiol, Berlin, Germany (Table 2) and primer efficiencies were tested. The normalized expression was calculated using the following efficiency corrected equation according to Simon [63]:

$$1)\;\mathrm{Normalized}\;\mathrm{expression}\;\left(\mathrm{NE}\right)=\;\frac{{\left(\mathrm{Efficiency}\;18S\right)}^{\;\mathrm{CT}\;18S}}{{\left(\mathrm{Efficiency}\;target\right)}^{\;\mathrm{CT}\;target}}$$

The normalized gene expression is given as fold gene expression to the intact tendons as calculated below:

$$2)\;\mathrm{Fold}\;\mathrm{gene}\;\mathrm{expression}=\;\frac{\mathrm{NE}\;pathologic\;tendons}{\mathrm{Mean}\;\mathrm{NE}\;intact\;tendons\;}$$

### 4.5. Statistics

Statistical analysis was performed using GraphPad Prism Version 7.0 (GraphPad Software, San Diego, CA, USA). For qRT-PCR analysis, 4 groups were compared (intact, tendinopathy, chronic ruptures and acute ruptures) and for histological evaluation 3 groups were compared (tendinopathy, chronic ruptures and acute ruptures). ANOVA using the Kruskal Wallis Test was performed to analyze significant differences between all groups. Dunn’s Multiple Comparison test was used for individual group comparison between either the intact group with the three tendon pathology groups or the three tendon pathology groups with each other. A *p* ≤ 0.05 was considered as statistically significant.

## 5. Conclusions

To sum up the present findings, the disturbed matrix metabolism, which is seen histologically, as well as by an imbalanced MMP/TIMP expression and highly increased collagen expression, seems to be the most important characteristic in tendinopathy and chronic ruptured tendons. Other mechanisms such as inflammation and innervation are indeed present in tendinopathy and chronic ruptured tendons, as indicated by CD45 and CD68 positive cells in the histological sections and the expression of inflammatory cytokines and nerve markers, but seem to play a more important role in the acute rupture situation. Targeting the disturbed matrix metabolism in chronic tendon pathologies might be a helpful tool for improved treatment strategies in the future.

## Figures and Tables

**Table 1:** Donor demographics.

Group	*n*-Value	Age (Mean ± SD)	BMI (Mean ± SD)	Sex (Female/Male)
Tendinopathy	7	45.3 ± 7.9 years	26.8 ± 5.2	3/4
Chronic Rupture	6	52.2 ± 12.6 years *	28.6 ± 5.2	1/5
Acute Rupture	13	32.0 ± 3.1 years *	23.5 ± 1.9	2/11
Intact (patient)	4	37.8 ± 12.8 years	30.5 ± 3.5	2/2
Intact (cadaver)	5	83.4 ± 2.9 years	-	3/2

* Significantly different (*p* = 0.005).

**Table 2:** qRT-PCR Primer.

Gene	Accession No.	Primer Sequence
*18S rRNA*	NM_022551	Forward: 5′ CGGAAAATAGCCTTTGCCATC 3′
		Reverse: 5′ AGTTCTCCCGCCCTCTTGGT 3′
*COL1A1*	NM_000088.3	Forward: 5′ TGACCTCAAGATGTGCCACT 3′
		Reverse: 5′ ACCAGACATGCCTCTTGTCC 3′
*COL3A1*	NM_000090.3	Forward: 5′ GCTGGCATCAAAGGACATCG 3′
		Reverse: 5′ TGTTACCTCGAGGCCCTGGT 3′
*COL5A2*	NM_000393	Forward: 5′ AGAAGCCTCCCAGAACATCA 3′
		Reverse: 5′ ACAGTCTTGCCCACATTTCC 3′
*MMP1*	NM_002421.3	Forward: 5′ CACGCCAGATTTGCCAAGAG 3′
		Reverse: 5′ GTCCCGATGATCTCCCCTGA 3′
*MMP2*	NM_ 004530	Forward: 5′ TGGATGATGCCTTTGCTCGT 3′
		Reverse: 5′ CCAGGAGTCCGTCCTTACCG 3′
*MMP3*	NM_002422.3	Forward: 5′ TGGGCCAGGGATTAATGGAG 3′
		Reverse: 5′ GGCCAATTTCATGAGCAGCA 3′
*MMP9*	NM_004994.2	Forward: 5′ GGGACGCAGACATCGTCATC 3′
		Reverse: 5′ GGGACCACAACTCGTCATCG 3′
*MMP10*	NM_ 002425	Forward: 5′ CCACCTGGACCTGGGCTTTA 3′
		Reverse: 5′ GAACTGGGCGAGCTCTGTGA 3′
*MMP13*	NM_002427.3	Forward: 5′ CCTTCCCAGTGGTGGTGATG 3′
		Reverse: 5′ CGGAGCCTCTCAGTCATGGA 3′
*TIMP1*	NM_003254.2	Forward: 5′ TTGGCTGTGAGGAATGCACA 3′
		Reverse: 5′ AAGGTGACGGGACTGGAAGC 3′
*TIMP2*	NM_003255.4	Forward: 5′ CCTGAGCACCACCCAGAAGA 3′
		Reverse: 5′ TCCATCCAGAGGCACTCGTC 3′
*TIMP3*	NM_000362.4	Forward: 5′ CCGAGGCTTCACCAAGATGC 3′
		Reverse: 5′ GCCATCATAGACGCGACCTG 3′
*TIMP4*	NM_003256.3	Forward: 5′ GAAGCCAACAGCCAGAAGCA 3′
		Reverse: 5′ TTCCCTCTGCACCAAGGACA 3′
*TNMD*	NM_022144.2	Forward: 5′ TTGAAGACCCACGAAGTAGA 3′
		Reverse: 5′ ATGACATGGAGCACACTTTC 3′
*IL33*	NM_033439	Forward: 5′ CCAACAGAAGGCCAAAGAAG 3′
		Reverse: 5′ AAGGCAAAGCACTCCACAGT 3′
*sST2*	NM_003856	Forward: 5′ CAACAAGAGGAAGGGCAAAA 3′
		Reverse: 5′ CAAATTCAGGGCCAGACAGT 3′
*IL1β*	NM_000576	Forward: 5′ TCCAGGAGAATGACCTGAGC 3′
		Reverse: 5′ GTGATCGTACAGGTGCATCG 3′
*TNFα*	NM_000594	Forward: 5′ AGCCCATGTTGTAGCAAACC 3′
		Reverse: 5′ GAGGTACAGGCCCTCTGATG 3′
*IL6*	NM_000600	Forward: 5′ TGAGGAGACTTGCCTGGTGA 3′
		Reverse: 5′ TTGGGTCAGGGGTGGTTATT 3′
*IL10*	NM_000572	Forward: 5′ TGAGAACAGCTGCACCCACT 3′
		Reverse: 5′ GGCAACCCAGGTAACCCTTA 3′
*TGFβ1*	NM_000660.4	Forward: 5′ AAGGACCTCGGCTGGAAGTG 3′
		Reverse: 5′ AGGGCCAGGACCTTGCTGTA 3′
*CD3*	NM_000073	Forward: 5′ CTGGGAAGTAATGCCAAGGA 3′
		Reverse: 5′ CCAACAGCAAGGACGAAAAT 3′
*CD68*	NM_001040059	Forward: 5′ CCACCTGCTTCTCTCATTCC 3′
		Reverse: 5′ ATTGTACTCCACCGCCATGT 3′
*CD80*	NM_005191	Forward: 5′ GCAGGGAACATCACCATCCA 3′
		Reverse: 5′ CAGGACAGCGTTGCCACTTC 3′
*CD206*	NM_002438	Forward: 5′ ACTGGGGCCAAGCTTCTCTG 3′
		Reverse: 5′ CACAGCCACGTCCCTTCAAC 3′
*MIF*	NM_002415	Forward: 5′ GGTTCCTCTCCGAGCTCACC 3′
		Reverse: 5′ TAGACCCTGTCCGGGCTGAT 3′
*PGP9.5*	NM_004181	Forward: 5′ CCATACAGGCAGCCCATGAT 3′
		Reverse: 5′ AGACCTTGGCAGCGTCCTTC 3′
*GAP43*	NM_002045	Forward: 5′ CCGGCAAAGCAGGAGAAACT 3′
		Reverse: 5′ TGGAGGACGGCGAGTTATCA 3′
*COX2*	NM_000963	Forward: 5′ TAGAGCCCTTCCTCCTGTGC 3′
		Reverse: 5′ TGGGGATCAGGGATGAACTT 3′
*FABP4*	NM_001442	Forward: 5′ ACTGGGCCAGGAATTTGACG 3′
		Reverse: 5′ ATGACGCATTCCACCACCAG 3′
*PPARγ*	NM_015869	Forward: 5′ AAAGTCCTTCCCGCTGACCA 3′
		Reverse: 5′ GGCCACCTCTTTGCTCTGCT 3′
*ADIPOQ*	NM_004797	Forward: 5′ TGACCAGGAAACCACGACTCA 3′
		Reverse: 5′ CCGATGTCTCCCTTAGGACCA 3′
*CEBPα*	NM_004364	Forward: 5′ AAGGCCAAGAAGTCGGTGGA 3′
		Reverse: 5′ GGCGGTCATTGTCACTGGTC 3′
*SCX*	Commercial Quantitect primer Assay Hs_SCXB_2_SG	

## References

[1] Sobhani S., Dekker R., Postema K., Dijkstra P.U. (2013). Epidemiology of ankle and foot overuse injuries in sports: A systematic review. Scand. J. Med. Sci. Sports.

[2] Khan K.M., Cook J.L., Bonar F., Harcourt P., Astrom M. (1999). Histopathology of common tendinopathies. Update and implications for clinical management. Sports Med..

[3] Maffulli N., Longo U.G., Denaro V. (2010). Novel approaches for the management of tendinopathy. J. Bone Jt. Surg. Am..

[4] Leung J.L., Griffith J.F. (2008). Sonography of chronic Achilles tendinopathy: A case-control study. J. Clin. Ultrasound.

[5] Longo U.G., Ronga M., Maffulli N. (2009). Achilles tendinopathy. Sports Med. Arthrosc..

[6] Maffulli N., Longo U.G., Franceschi F., Rabitti C., Denaro V. (2008). Movin and Bonar scores assess the same characteristics of tendon histology. Clin. Orthop. Relat. Res..

[7] Maffulli N., Longo U.G., Maffulli G.D., Rabitti C., Khanna A., Denaro V. (2011). Marked pathological changes proximal and distal to the site of rupture in acute Achilles tendon ruptures. Knee Surg. Sports Traumatol. Arthrosc..

[8] Pingel J., Lu Y., Starborg T., Fredberg U., Langberg H., Nedergaard A., Weis M., Eyre D., Kjaer M., Kadler K.E. (2014). 3-D ultrastructure and collagen composition of healthy and overloaded human tendon: Evidence of tenocyte and matrix buckling. J. Anat..

[9] Movin T., Gad A., Reinholt F.P., Rolf C. (1997). Tendon pathology in long-standing achillodynia. Biopsy findings in 40 patients. Acta Orthop. Scand..

[10] Fu S.C., Chan B.P., Wang W., Pau H.M., Chan K.M., Rolf C.G. (2002). Increased expression of matrix metalloproteinase 1 (MMP1) in 11 patients with patellar tendinosis. Acta Orthop. Scand..

[11] Raleigh S.M., van der Merwe L., Ribbans W.J., Smith R.K., Schwellnus M.P., Collins M. (2009). Variants within the *MMP3* gene are associated with Achilles tendinopathy: Possible interaction with the *COL5A1* gene. Br. J. Sports Med..

[12] Ireland D., Harrall R., Curry V., Holloway G., Hackney R., Hazleman B., Riley G. (2001). Multiple changes in gene expression in chronic human Achilles tendinopathy. Matrix Biol..

[13] Alfredson H., Lorentzon M., Backman S., Backman A., Lerner U.H. (2003). cDNA-arrays and real-time quantitative PCR techniques in the investigation of chronic Achilles tendinosis. J. Orthop. Res..

[14] Battery L., Maffulli N. (2011). Inflammation in overuse tendon injuries. Sports Med. Arthrosc..

[15] Abate M., Silbernagel K.G., Siljeholm C., di Iorio A., de Amicis D., Salini V., Werner S., Paganelli R. (2009). Pathogenesis of tendinopathies: Inflammation or degeneration?. Arthritis Res. Ther..

[16] Del Buono A., Battery L., Denaro V., Maccauro G., Maffulli N. (2011). Tendinopathy and inflammation: Some truths. Int. J. Immunopathol. Pharmacol..

[17] Fredberg U., Stengaard-Pedersen K. (2008). Chronic tendinopathy tissue pathology, pain mechanisms, and etiology with a special focus on inflammation. Scand. J. Med. Sci. Sports.

[18] Kannus P., Jozsa L. (1991). Histopathological changes preceding spontaneous rupture of a tendon. A controlled study of 891 patients. J. Bone Jt. Surg. Am..

[19] Schubert T.E., Weidler C., Lerch K., Hofstadter F., Straub R.H. (2005). Achilles tendinosis is associated with sprouting of substance P positive nerve fibres. Ann. Rheum. Dis..

[20] Scott A., Lian O., Bahr R., Hart D.A., Duronio V., Khan K.M. (2008). Increased mast cell numbers in human patellar tendinosis: Correlation with symptom duration and vascular hyperplasia. Br. J. Sports Med..

[21] Behzad H., Sharma A., Mousavizadeh R., Lu A., Scott A. (2013). Mast cells exert pro-inflammatory effects of relevance to the pathophyisology of tendinopathy. Arthritis Res. Ther..

[22] Kragsnaes M.S., Fredberg U., Stribolt K., Kjaer S.G., Bendix K., Ellingsen T. (2014). Stereological quantification of immune-competent cells in baseline biopsy specimens from achilles tendons: Results from patients with chronic tendinopathy followed for more than 4 years. Am. J. Sports Med..

[23] Millar N.L., Hueber A.J., Reilly J.H., Xu Y., Fazzi U.G., Murrell G.A., McInnes I.B. (2010). Inflammation is present in early human tendinopathy. Am. J. Sports Med..

[24] Dakin S.G., Newton J., Martinez F.O., Hedley R., Gwilym S., Jones N., Reid H.A.B., Wood S., Wells G., Appleton L. (2017). Chronic inflammation is a feature of Achilles tendinopathy and rupture. Br. J. Sports Med..

[25] Rees J.D., Maffulli N., Cook J. (2009). Management of tendinopathy. Am. J. Sports Med..

[26] Alfredson H., Lorentzon R. (2002). Chronic tendon pain: No signs of chemical inflammation but high concentrations of the neurotransmitter glutamate. Implications for treatment?. Curr. Drug Targets.

[27] Dean B.J., Snelling S.J., Dakin S.G., Murphy R.J., Javaid M.K., Carr A.J. (2015). Differences in glutamate receptors and inflammatory cell numbers are associated with the resolution of pain in human rotator cuff tendinopathy. Arthritis Res. Ther..

[28] Spang C., Harandi V.M., Alfredson H., Forsgren S. (2015). Marked innervation but also signs of nerve degeneration in between the Achilles and plantaris tendons and presence of innervation within the plantaris tendon in midportion Achilles tendinopathy. J. Musculoskelet. Neuronal Interact..

[29] Khan K.M., Cook J.L., Maffulli N., Kannus P. (2000). Where is the pain coming from in tendinopathy? It may be biochemical, not only structural, in origin. Br. J. Sports Med..

[30] Legerlotz K., Jones E.R., Screen H.R., Riley G.P. (2012). Increased expression of IL-6 family members in tendon pathology. Rheumatology.

[31] Burssens P., Forsyth R., Steyaert A., Van Ovost E., Praet M., Verdonk R. (2005). Influence of burst TENS stimulation on collagen formation after Achilles tendon suture in man. A histological evaluation with Movat’s pentachrome stain. Acta Orthop. Belg..

[32] Pingel J., Fredberg U., Qvortrup K., Larsen J.O., Schjerling P., Heinemeier K., Kjaer M., Langberg H. (2012). Local biochemical and morphological differences in human Achilles tendinopathy: A case control study. BMC Musculoskelet. Disord..

[33] Goncalves-Neto J., Witzel S.S., Teodoro W.R., Carvalho-Junior A.E., Fernandes T.D., Yoshinari H.H. (2002). Changes in collagen matrix composition in human posterior tibial tendon dysfunction. Jt. Bone Spine.

[34] September A.V., Schwellnus M.P., Collins M. (2007). Tendon and ligament injuries: The genetic component. Br. J. Sports Med..

[35] Magra M., Maffulli N. (2008). Genetic aspects of tendinopathy. J. Sci. Med. Sport.

[36] Mokone G.G., Schwellnus M.P., Noakes T.D., Collins M. (2006). The *COL5A1* gene and Achilles tendon pathology. Scand. J. Med. Sci. Sports.

[37] Shukunami C., Takimoto A., Oro M., Hiraki Y. (2006). Scleraxis positively regulates the expression of tenomodulin, a differentiation marker of tenocytes. Dev. Biol..

[38] Docheva D., Hunziker E.B., Fassler R., Brandau O. (2005). Tenomodulin is necessary for tenocyte proliferation and tendon maturation. Mol. Cell Biol..

[39] Millar N.L., Gilchrist D.S., Akbar M., Reilly J.H., Kerr S.C., Campbell A.L., Murrell G.A., Liew F.Y., Kurowska-Stolarska M., McInnes I.B. (2015). MicroRNA29a regulates IL-33-mediated tissue remodelling in tendon disease. Nat. Commun..

[40] Sharma P., Maffulli N. (2006). Biology of tendon injury: Healing, modeling and remodeling. J. Musculoskelet. Neuronal Interact..

[41] Del Buono A., Oliva F., Longo U.G., Rodeo S.A., Orchard J., Denaro V., Maffulli N. (2012). Metalloproteases and rotator cuff disease. J. Shoulder Elbow Surg..

[42] Riley G.P., Curry V., DeGroot J., van El B., Verzijl N., Hazleman B.L., Bank R.A. (2002). Matrix metalloproteinase activities and their relationship with collagen remodelling in tendon pathology. Matrix Biol..

[43] Klatte-Schulz F., Aleyt T., Pauly S., Geissler S., Gerhardt C., Scheibel M., Wildemann B. (2015). Do Matrix Metalloproteases and Tissue Inhibitors of Metalloproteases in Tenocytes of the Rotator Cuff Differ with Varying Donor Characteristics?. Int. J. Mol. Sci..

[44] Minkwitz S., Schmock A., Kurtoglu A., Tsitsilonis S., Manegold S., Wildemann B., Klatte-Schulz F. (2017). Time-Dependent Alterations of MMPs, TIMPs and Tendon Structure in Human Achilles Tendons after Acute Rupture. Int. J. Mol. Sci..

[45] Jones S.A. (2005). Directing transition from innate to acquired immunity: Defining a role for IL-6. J. Immunol..

[46] Lin T.W., Cardenas L., Glaser D.L., Soslowsky L.J. (2006). Tendon healing in interleukin-4 and interleukin-6 knockout mice. J. Biomech..

[47] Docheva D., Muller S.A., Majewski M., Evans C.H. (2015). Biologics for tendon repair. Adv. Drug Deliv. Rev..

[48] Scheller J., Chalaris A., Schmidt-Arras D., Rose-John S. (2011). The pro- and anti-inflammatory properties of the cytokine interleukin-6. Biochim. Biophys. Acta.

[49] Durgam S., Stewart M. (2017). Cellular and Molecular Factors Influencing Tendon Repair. Tissue Eng. Part B Rev..

[50] Dakin S.G., Werling D., Hibbert A., Abayasekara D.R., Young N.J., Smith R.K., Dudhia J. (2012). Macrophage sub-populations and the lipoxin A4 receptor implicate active inflammation during equine tendon repair. PLoS ONE.

[51] Stolk M., Klatte-Schulz F., Schmock A., Minkwitz S., Wildemann B., Seifert M. (2017). New insights into tenocyte-immune cell interplay in an in vitro model of inflammation. Sci. Rep..

[52] Klatte-Schulz F., Gerhardt C., Scheibel M., Wildemann B., Pauly S. (2014). Relationship between muscle fatty infiltration and the biological characteristics and stimulation potential of tenocytes from rotator cuff tears. J. Orthop. Res..

[53] Klatte-Schulz F., Pauly S., Scheibel M., Greiner S., Gerhardt C., Hartwig J., Schmidmaier G., Wildemann B. (2013). Characteristics and stimulation potential with BMP-2 and BMP-7 of tenocyte-like cells isolated from the rotator cuff of female donors. PLoS ONE.

[54] Klatte-Schulz F., Pauly S., Scheibel M., Greiner S., Gerhardt C., Schmidmaier G., Wildemann B. (2012). Influence of age on the cell biological characteristics and the stimulation potential of male human tenocyte-like cells. Eur. Cell Mater..

[55] Pingel J., Petersen M.C., Fredberg U., Kjaer S.G., Quistorff B., Langberg H., Hansen J.B. (2015). Inflammatory and Metabolic Alterations of Kager’s Fat Pad in Chronic Achilles Tendinopathy. PLoS ONE.

[56] Fain J.N. (2006). Release of interleukins and other inflammatory cytokines by human adipose tissue is enhanced in obesity and primarily due to the nonfat cells. Vitam. Horm..

[57] Gomez R., Lago F., Gomez-Reino J., Dieguez C., Gualillo O. (2009). Adipokines in the skeleton: Influence on cartilage function and joint degenerative diseases. J. Mol. Endocrinol..

[58] Nishio Y., Nishihira J., Ishibashi T., Kato H., Minami A. (2002). Role of macrophage migration inhibitory factor (MIF) in peripheral nerve regeneration: Anti-MIF antibody induces delay of nerve regeneration and the apoptosis of Schwann cells. Mol. Med..

[59] Kendal A., Snelling S., Dakin S., Stace E., Mouthuy P.A., Carr A. (2017). Resorbable electrospun polydioxanone fibres modify the behaviour of cells from both healthy and diseased human tendons. Eur. Cell Mater..

[60] Nissinen L., Kahari V.M. (2014). Matrix metalloproteinases in inflammation. Biochim. Biophys. Acta.

[61] Itaya T., Kagami H., Okada K., Yamawaki A., Narita Y., Inoue M., Sumita Y., Ueda M. (2009). Characteristic changes of periodontal ligament-derived cells during passage. J. Periodontal Res..

[62] Brophy R.H., Rai M.F., Zhang Z., Torgomyan A., Sandell L.J. (2012). Molecular analysis of age and sex-related gene expression in meniscal tears with and without a concomitant anterior cruciate ligament tear. J. Bone Jt. Surg. Am..

[63] Simon P. (2003). Q-Gene: Processing quantitative real-time RT-PCR data. Bioinformatics.

